# Biallelic *ANGPT2* loss-of-function causes severe early-onset non-immune hydrops fetalis

**DOI:** 10.1136/jmedgenet-2021-108179

**Published:** 2021-12-07

**Authors:** Marie F. Smeland, Pascal Brouillard, Trine Prescott, Laurence M Boon, Bodil Hvingel, Cecilie V Nordbakken, Mona Nystad, Øystein L. Holla, Miikka Vikkula

**Affiliations:** 1 Department of Medical Genetics, University Hospital of North Norway, Tromsø, Norway; 2 Human Molecular Genetics, de Duve Institute, Universite catholique de Louvain, Brussels, Belgium; 3 Department of Medical Genetics, Telemark Hospital, Skien, Norway; 4 Center for Vascular Anomalies, Division of Plastic Surgery, VASCERN VASCA European Reference Centre, University Hospital Saint-Luc, Bruxelles, Belgium; 5 Department of Obstetrics and Gynecology, University Hospital of North Norway, Tromsø, Norway; 6 Department of Clinical Pathology, University Hospital of North Norway, Tromsø, Norway; 7 Department of Clinical Medicine, University Hospital of North Norway, Tromsø, Norway

**Keywords:** genetics, medical, loss of function mutation, female urogenital diseases and pregnancy complications

## Abstract

**Background:**

Hydrops fetalis, a pathological fluid accumulation in two or more body compartments, is aetiologically heterogeneous. We investigated a consanguineous family with recurrent pregnancy loss due to severe early-onset non-immune hydrops fetalis.

**Methods and results:**

Whole exome sequencing in four fetuses with hydrops fetalis revealed that they were homozygous for the angiopoietin-2 (*ANGPT2*) variant Chr8 (GRCh37/Hg19): 6385085T>C, NM_001147.2:c.557A>G. The substitution introduces a cryptic, exonic splice site predicted to result in loss of 10 nucleotides with subsequent shift in reading frame, leading to a premature stop codon. RNA analysis in the heterozygous parents demonstrated loss of detectable mutant allele, indicative of loss-of-function via nonsense-mediated mRNA decay. Serum ANGPT2 levels were reduced in the parents. In a pregnancy with a healthy, heterozygous child, transiently increased fetal nuchal translucency was noted.

**Conclusion:**

Pathogenic heterozygous *ANGPT2* missense variants were recently shown to cause autosomal dominant primary lymphoedema. *ANGPT2* is a ligand of the TIE1-TIE2 (tyrosine kinase with immunoglobulin-like and epidermal growth factor-like domains 1 and 2) pathway. It is critical to the formation and remodelling of blood and lymphatic vessels and is involved in vessel maintenance. ANGPT2 knockout mice die from generalised lymphatic dysfunction. We show here that a homozygous pathogenic variant causes loss-of-function and results in severe early-onset hydrops fetalis. This is the first report of an autosomal recessive *ANGPT2*-related disorder in humans.

## Introduction

Hydrops fetalis is a pathological accumulation of fluid in two or more body compartments.^1^ This aetiologically heterogeneous condition is caused by increased interstitial fluid production or decreased lymphatic return,^2^ and may result in increased nuchal translucency, lymphatic malformations (eg, cystic hygroma), subcutaneous fluid accumulation, pleural/pericardial effusion, ascites and placental thickening.

Historically, red cell allo-immunisation was the major cause of hydrops fetalis. Today, non-immune hydrops fetalis (NIHF; Mendelian Inheritance in Man, MIM: 236750) constitutes approximately 90% of cases.^3^ The estimated prevalence of hydrops fetalis ranges from 1:1500 to 1:4000 births.^4^ Early onset and aetiology are prognostic predictors in NIHF. Presentation in the first trimester increases the likelihood of a fetal aneuploidy. In a prenatal series of more than 12 000 pregnancies, almost half of 66 NIHF cases were attributed to a chromosomal abnormality.^5^ Aneuploidy conferred an extremely poor prognosis.^5^ In liveborn infants with NIHF, neonatal mortality is as high as 60%.^6^

Bellini *et al* 7 presented a classification scheme for NIHF based on underlying mechanism, including chromosomal imbalance, infection, placental abnormality, twin-to-twin transfusion, cardiovascular disorders, syndromes and lymphatic dysplasia. In an update, they classified 19.8% of cases as idiopathic.^8^ In a recent review including only cases meeting the strict criteria for NIHF, the aetiology was identified in 86.3%.^9^ The most prevalent aetiologies in the study by Laterre *et al* 9 were chromosomal imbalance (33 of 102; 32.4%), lymphatic dysplasia (14 of 102; 13.7%) and cardiovascular disorders (10 of 102; 9.8%). The authors also developed a flow chart outlining a systematic diagnostic approach that emphasises the importance of next-generation sequencing (NGS).^9^

In a series of 127 cases of prenatally diagnosed NIHF, which were aetiologically unresolved by standard analyses, a genetic cause was detected in 29% by whole exome sequencing (WES).^10^ A comprehensive systematic review of monogenic causes of NIHF identified 517 candidate genes, including 177 for which evidence was strong or emerging.^11^ Of these 177, 5.1% were lymphatic-related genes. Autosomal recessive inheritance was the most frequent (61.8%) when aetiology was known, although all Mendelian modes of inheritance were represented.^11^

NIHF due to lymphatic abnormalities is a form of generalised lymphatic dysplasia, a subgroup of primary lymphoedema.^12^ In Genomics England’s PanelApp, evidence of an association between pathogenic variants in 40 genes and primary lymphoedema is strong (‘green’ category genes; panel version: 2.12, 13 March 2021). For fetal hydrops (panel version 1.25, 13 March 2021), the evidence for causation is strong for 56 genes.^13^ The two panels overlap with 19 genes.

Subsequent to the original publication of primary lymphoedema involving the vascular endothelial growth factor 3 gene (VEGFR3, encoded by the *FLT4* gene),^14 15^ the central role of VEGFR3 in signalling was identified.^16^ Genes more recently added to the pathway include *ADAMTS3*,^17^ *FBXL7* 18 and *ARAF.* 19 New pathways involving lymphangiogenesis and remodelling have also been identified and involve genes such as *PIEZO1*,^20^ *EPHB4*,^21^ *CALCR* 22 and *GDF2*.^23^

In 2020, heterozygous missense mutations in angiopoietin-2 (*ANGPT2*) were reported to cause primary lymphoedema with reduced penetrance and variable expressivity in five unrelated families,^24^ implicating another endothelial growth factor receptor pathway, the ANGPT2–TIE2 tyrosine kinase pathway. In these families, lymphoedema was not severe and tended to resolve during childhood.

ANGPT2 disrupts angiogenesis in the developing embryo by antagonising the effects of angiopoietin-1 and Tie2.^25^ In mice, homozygous deletion of *ANGPT2* or treatment with Angpt2-blocking antibodies results in morphologically and functionally abnormal lymphatics and neonatal death.^26^ Pathogenic *ANGPT2* variants identified in lymphoedema in humans and studied in mice affect protein function via haploinsufficiency or a dominant-negative mechanism. The resultant reduced ANGPT2 secretion or ANGPT2 binding to integrins causes dysplastic lymphatic vasculature in murine ear skin.^24^

Here we report a consanguineous couple with at least four fetuses with severe early-onset NIHF and homozygosity for a loss-of-function (LOF) variant in *ANGPT2*. This work underscores the pivotal role of *ANGPT2* in lymphatic development and adds to the growing list of genes associated with NIHF.

## Patients and methods

### Methods

#### Nucleic acid extraction

DNA was extracted from EDTA blood with EZ1 DSP DNA Blood Kit using an EZ1 Advanced XL (QIAGEN) from amniocytes (for WES) and chorion villus biopsies (for SNP array analysis) using QiaSymphony (QIAGEN) or QIAamp DNA Micro Kit (QIAGEN). For quantitative fluorescent (QF)-PCR analysis of amniocytes and chorion villus biopsies, rapid DNA extraction was performed using InstaGene Matrix Chelex 100 (Bio-Rad, California, USA).

RNA was extracted from whole blood using TRIzol reagent (Invitrogen, California, USA) and retrotranscribed with moloney murine leukaemia virus RT (ThermoFisher, Massachusetts, USA). For RT-PCR, primers were chosen in exons distant from those carrying the pathogenic variant NM_001147.2: c.557A>G and the benign marker variant NM_001147.2:c.882G>A (sequences available on request). RNA was also isolated from cultured fibroblasts derived from a paternal skin biopsy.

#### ELISA measurements of ANGPT2

ANGPT2 protein levels were measured in blood-EDTA plasma using the Human Angiopoietin-2 Quantikine ELISA Kit (R&D Systems, Minnesota, USA), according to the manufacturer’s protocol.

#### Whole exome sequencing

Nextera Rapid Capture Exome sample prep and enrichment (Illumina, California, USA) were carried out according to the manufacturer’s instructions. Libraries were sequenced 2×150 bp on a NextSeq500 (Illumina). Bcl2fastq2 conversion software (Illumina) was used for de-multiplexing. Reads were aligned to reference genome GRCh37/hg19 using Burrows-Wheeler Aligner, BWA.^8^ Picard was used to remove PCR duplicates.^27^ The Genome Analysis Toolkit was applied for base quality score recalibration, insertion and deletion (INDEL) realignment, and single nucleotide variant and INDEL discovery. Sequence variants were annotated with Annovar.^28^ Regions of homozygosity were detected using BCFtools/Samtools. Filtus software was used for bioinformatic filtering of variants.^29^ Integrative Genomics Viewer was used for NGS data visualisation.^30^ Splice site predictions were performed using SpliceSiteFinder, MaxEntScan, NNSPLICE, GeneSplicer and Human Splicing Finder through Alamut Visual software (Interactive Biosoftware, Rouen, France).

Samples from both parents, four affected fetuses and one clinically unaffected child were exome-sequenced. *ANGPT2* was analysed by NGS in a blood sample from the maternal aunt (II-6) as a control of her Sanger sequencing result.

#### Sanger sequencing

DNA from samples II-6 (including a control sample) and III-9 were Sanger-sequenced on an ABI 3130xl using standard protocols to determine *ANGPT2* genotype. For the other samples, Sanger sequencing was used to verify *ANGPT2* results from NGS.

#### QF–PCR (III-4, III-5 and III-7)

QF-PCR/rapid aneuploidy diagnostics test was performed with the Elucigene QST*Rplusv2 Kit (Elucigene Diagnostics, Manchester, England). Fragments were analysed using capillary electrophoresis on ABI 3500xl Genetic Analyzer (Applied Biosystems, California, USA) with accompanying GeneMapper software according to the manufacturer’s protocols.

#### Standard G-banding/karyotyping of amniocytes and parental blood samples (III-4, II-1 and II-2)

G-banding of metaphase chromosomes was performed on amniocytes from fetus III-4 and sodium-heparin peripheral blood samples from the parents (II-1 and II-2) according to standard protocols.

#### SNP array analysis (III-4, III-5 and III-7)

Molecular karyotyping of DNA extracted from amniocytes and chorion villi was performed using SNP array analysis (CytoScan HD; Affymetrix, California, USA). Filtering of data from SNP array analysis was performed using ChAS (Chromosome Analysis Suite) software (Affymetrix) and Cartagenia Bench Lab CNV (Agilent Technologies, California, USA). Positions refer to Genome Reference Consortium Human Genome Build 37 (GRCh37) (hg19). The following were the filter settings for analysis: 400 kb and 30 markers for loss, 90 markers for gains; 5 Mb and 50 SNP markers for regions of homozygosity.

#### Lymphohistochemical staining in postmortem samples (III-4 and III-5)

Lymphohistochemical staining in skin/subcutaneous tissue from the neck and lung was performed on formalin-fixed paraffin-embedded (FFPE) tissue sections with marker D2-40 (podoplanin) (Roche) for lymphatic endothelium.

#### Placental examination (III-4 and III-5)

Placental FFPE tissue was stained with H&E according to the manufacturer’s protocol to visualise the microanatomy of the placenta (nuclear components, cytoplasmic collagen and elastic fibres, muscle fibres and red blood cells).

## Results

### Clinical summary

**Figure 1:**
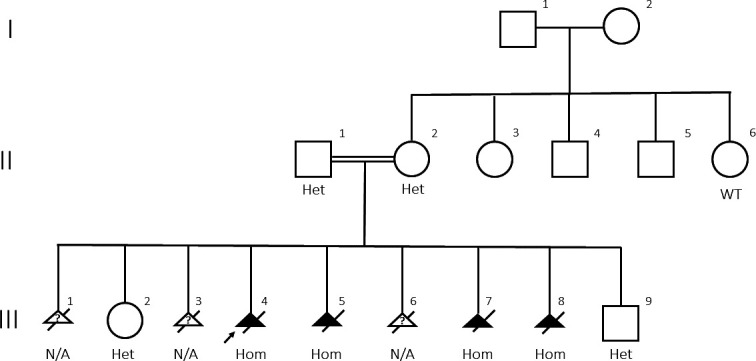
Pedigree of a family with ANGPT2 c.557A>G r.557_566del. Triangles in black are fetal losses with hydrops fetalis, and white triangles with question mark are early pregnancy losses not amenable to evaluation. Created by the authors. Het, heterozygous NM_001147.2:c.557A>G r.557_566del; Hom, homozygous NM_001147.2:c.557A>G r.557_566del; N/A, not available for genetic analysis; WT, wild-type, homozygous NM_001147.2:c.557A r.557A.

The clinical and molecular findings are summarised in table 1 and figure 1.

**Table 1:** Clinical information of the members of a family with *ANGPT2* c.557A>G r.557_566del

Individual/pregnancy	III-3	III-4	III-5	III-7	III-8	III-9	II-1	II-2	II-6
Phenotype	Healthy girl	IUFD, week 24+2	IUFD, week 27+3	TOP, week 14	Miscarriage, week 14	Healthy boy	Healthy father	Healthy mother	Maternal aunt
Sex	Female	Male	Female	Male	Male	Male	Male	Female	Female
Sample/analysis	Blood	Amnion	CVS	Amnion	Fibroblasts	Umbilical cord	Blood	Blood	Blood
Analysis	WES	WES	WES	WES	WES	Sanger	WES	WES	Sanger, WES
Genotype	Het	Hom	Hom	Hom	Hom	Het	Het	Het	WT/WT
Outcome	Healthy.	Severe, progressive hydrops, IUFD week 24.	Severe, progressive hydrops, IUFD week 27.	Early detected severe hydrops, TOP week 14.	Early detected severe hydrops, spontaneous abortion week 14.	Healthy.	Healthy.	Healthy.	‘Oedema’ in childhood, lower extremity lymphoedema.
Early US, including NT	Week 11+2 normal, no measurement of NT.	Week 11 normal, no measurement of NT.	Week 12+3 NT 5.0, cystic hygroma subcutaneous fluid.	Week 12+4 cystic hygroma, subcutaneous fluid.	Week 11+0 NT 3.7, generalised subcutaneous fluid, cystic hygroma.	Week 12+4 NT 4.5	–	–	–
Later US	Week 18 normal. Week 32 normal.	Week 19+5 (LMP). Week 17 (US) screening hydrothorax, ascites, subcutaneous fluid.	Week 16 hydrothorax, subcutaneous fluid, cystic hygroma. Week 26 ascites.	Week 14+4 cystic hygroma and hydrothorax.	Week 12+1 NT 6–7 mm.	Week 14+2 NT normal.	–	–	–
PM	NA	Yes	Yes	NA	NA	NA	–	–	–
Cardiac	NA	Tetralogy of Fallot	Normal at PM	NA	NA	NA	–	–	–
Skeletal/growth	NA	2-week discrepancy EDD from LMP and from US.	Short long bones, bell-shaped thorax (due to hydrothorax/hypoplastic lungs.	NA	NA	NA	–	–	–
IHC in lymphatic endothelium	NA	No observed increase in number of lymphatic vessels	No observed increase in number of lymphatic vessels	NA	NA	NA	–	–	–
Placenta	NA	Hydropic placenta. Thick (3 cm), oedematous. Weight 360 g.	Hydropic placenta. Thick (2,9 cm). Weight 440 g. Abnormal vascular architecture/proliferation of vessels.	NA	NA	NA	–	–	–

Created by the authors.

CVS, chorionic villous biopsy; EDD, estimated due date; Het, heterozygous; Hom, homozygous; IHC, immunohistochemistry; IUFD, intrauterine fetal death; LMP, last menstrual period; NA, not assessed/not applicable; NT, nuchal translucency; PM, postmortem examination; TOP, termination of pregnancy; US, ultrasound; WES, whole exome sequencing; WT, wild-type.

The healthy mother (II-1) and father (II-2) are second cousins once removed and originally from central Asia. She has polycystic ovary syndrome. Both parents have normal haemoglobin profiles and neither harbours an alpha-globin deletion. Maternal parvovirus serum IgG in the fifth pregnancy was indicative of previous infection. Screening for maternal erythrocyte and platelet antibodies was negative. Standard karyotyping (G-band analysis) of the parents was normal. There is no clinical suspicion of lymphoedema in either parent, although their prenatal and neonatal histories are not available.

The mother presented at age 26 years in the second trimester of her fourth pregnancy (III-4) due to fetal hydrops. Fetal ultrasound at 11+5 weeks had been reported as normal, although nuchal translucency was not assessed. Routine ultrasound at 19+5 weeks revealed a male fetus (III-4) with severe hydrops. Additional findings included gestational age by ultrasound at 17+4 weeks, no evidence of fetal anaemia based on cerebral artery Doppler, and negative rapid testing for trisomy 13, 18 and 21 and cytogenetic karyotyping in amniocytes. Maternal serology and PCR analysis of amniotic fluid detected no evidence of a viral infection. At 20 weeks, fluid accumulation was increased and tetralogy of Fallot was noted. Intrauterine death occurred at 26+3 weeks.

The mother’s first pregnancy ended in an early miscarriage (III-1).

The couple has a healthy daughter (III-2) born following a normal pregnancy in which an ultrasound at 11+2 weeks was reported as normal, although nuchal translucency was not assessed. She is now school-aged and there is no suspicion of oedema anamnestically.

The third pregnancy resulted in a miscarriage (III-3) at 9 weeks.

**Figure 2:**
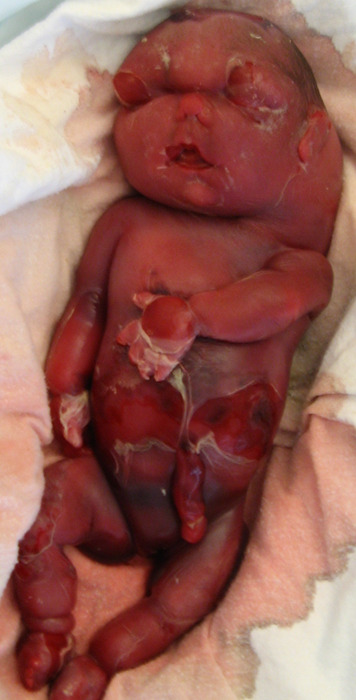
Photograph of a fetus (III-5) with intrauterine death at 27 gestational weeks. Note the severe subcutaneous oedemas, deformed facial features and large cystic hygroma of the neck. Created by the authors.

In the fifth pregnancy, severe hydrops including a large nuchal cyst was detected at 12 weeks in the female fetus (III-5). Results of rapid testing for trisomy 13, 18 and 21 and chromosomal microarray on DNA extracted from a chorionic villus biopsy were normal. The hydrops progressed and the couple was informed of a poor prognosis, including the risk of maternal mirror syndrome. Fetal demise occurred at 27 weeks (figure 2). Autopsy revealed severe hydrops with hydrothorax, hypoplastic lungs, a bell-shaped thorax, short long bones and a hydropic placenta.

The sixth pregnancy ended in a termination (III-6) after detection of a blighted ovum at 10+4 weeks.

The seventh pregnancy was terminated (III-7) at 15 weeks after detection of severe subcutaneous oedema and a cystic hygroma at 12+4 weeks. Rapid aneuploidy testing was normal. Chromosomal microarray was normal and demonstrated homozygosity stretches in common with fetuses III-4 and III-5, in keeping with parental consanguinity.

The eighth pregnancy ended in a miscarriage (III-8) at 14–15 weeks following detection of increased nuchal translucency (week 11+0, 3.7 mm, >99th centile) and severe generalised subcutaneous fluid accumulation.

In the ninth pregnancy (III-9), ultrasound at 12+2 gestational weeks showed increased nuchal translucency (4.5 mm, >99th centile) with a narrow brim of subcutaneous fluid extending to the upper back. However, at 14+2 weeks no abnormal fluid accumulation was seen. Pregnancy proceeded uneventfully and a healthy male infant was born at term. He is now 12 months old and there has been no suspicion of oedema at any time.

### Clinical assessment of additional family members

Both parents have several siblings, but only the maternal 30-year-old sister (II-6) has been available for investigation. She reported childhood ‘oedemas’, but her paediatric record is unavailable. In young adulthood, she presented with intermittent swelling of the ankles and feet, especially on the left. Lymphoscintigraphy of her lower extremities failed to visualise the lymphatic vessels or the inguinal nodes on the left. In the right leg, delayed clearance and several foci of increased uptake were interpreted as evidence of abnormal lymphatic vessels morphology.

### Whole exome sequencing

WES revealed rare, coding or splice site variants compatible with autosomal recessive inheritance in seven genes not previously been associated with hydrops fetalis (table 2). These were submitted to GeneMatcher.^31^ The only potentially relevant match was for *ANGPT2*, a case series with autosomal dominant lymphoedema, which was then unpublished.^24^ Subsequently, in a new pregnancy with an affected fetus (III-8), exome sequencing excluded all candidate genes except *ANGPT2*. The detected variant in our family, (GRCh37/Hg19): 8:6385085T>C, NM_001147.2:c.557A>G p.(Asp186Gly) in exon 3, was identified in the homozygous state in all investigated hydropic fetuses and in the heterozygous state in the unaffected parents (figure 1 and table 1). The *ANGPT2* variant was absent from gnomAD and predicted activation of a cryptic 5’ splice site between c.556 and c.557, leading to removal of 10 bp from the 3’ end of exon 3 and introduction of a premature termination codon: r.557_566del p.(Asp186Valdelfs*3).

**Table 2:** Candidate genes

Gene	Chromosome	Position	Reference allele	Alternative allele	cDNA change	Protein change	gnomAD popmax
*ANGPT2*	8	6385085	T	C	NM_001147.2:c.557A>G	p.(Asp186Gly)	0
*CYP2C19*	10	96535218	G	C	NM_000769.2:c.403G>C	p.(Gly135Arg)	0
*RRP12*	10	99118361	C	T	NM_015179.1:c.3724G>A	p.(Val1242Met)	20/30616 South Asian alleles
*LOXL4*	10	100016620	C	T	NM_032211.6:c.1345G>A	p.(Val449Met)	14/30616 South Asian alleles
*PYROXD2*	10	100154981	T	C	NM_032709.2:c.757A>G	p.(Met253Val)	18/30616 South Asian alleles
*SH3PXD2A*	10	105362591	G	A	NM_014631.1:c.2300C>T	p.(Ser767Phe)	9/30612 South Asian alleles
*TECTB*	10	114044422	A	T	NM_058222.2:c.206A>T	p.(Tyr69Phe)	5/30356 South Asian alleles (1 homozygote)

Whole exome sequencing of II-1, II-2, III-2, III-4, III-5 and III-7 revealed rare, coding variants in seven candidate genes which had not previously been associated with hydrops. All variants were entered in GeneMatcher. Sequencing of III-8 had not been undertaken at that time.

Created by the authors.

Individual II-6 did not carry *ANGPT2* c.557A>G. No pathogenic or likely pathogenic variants causative of monogenic lymphoedema were detected in the 40 genes subsequently analysed (PanelApp’s ‘green’ genes, primary lymphoedema; V.2.2, 6 June 2021).^13^

### Sanger sequencing

The unaffected brother (III-9) was heterozygous for NM_001147.2:c.557A>G, whereas the maternal aunt (II-6) did not carry the variant (a control sample was also analysed by Sanger sequencing and by WES).

### mRNA analyses

**Figure 3:**
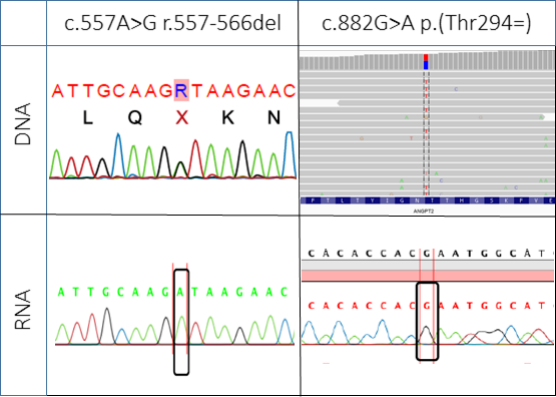
DNA and RNA analyses. Sequencing of the pathogenic variant *ANGPT2* c.557A>G (heterozygous, represented by an R) and the benign marker variant c.882 G>A (heterozygous C/T in the genomic minus strand alignment from WES) in DNA (top) and RNA isolated from lymphocytes from individual II-1 (bottom). RNA only contains one of the two alleles (WT for the mutation position). Created by the authors. WES, whole exome sequencing; WT, wild-type.

mRNA from the heterozygous parents showed only the wild-type (WT) (A) allele at position c.557 (figure 3). Loss of the allele carrying the likely pathogenic variant c.557A>G was confirmed by genotyping a second variant NM_001147.2:c.882G>A p.(Thr294=) in exon 5, heterozygously present in both parents’ DNA. Only the G-allele was observed in mRNA from fibroblasts and lymphocytes. Thus, both parents expressed only a single WT *ANGPT2* allele, in keeping with degradation of the mutant allele with a premature termination codon by nonsense-mediated mRNA decay.

To confirm that ANGPT2 expression was reduced, plasma concentrations were measured by ELISA. The heterozygous father’s plasma concentration was reduced (672.2 pg/mL) (table 3). This is similar to the level measured for the individual with the dominant-negative acting *ANGPT2* variant R492Q reported by Leppänen *et al*.^24^ The heterozygous mother was pregnant at the time of sampling. Her serum concentration of ANGPT2 (8294 pg/mL) was increased compared with non-pregnant controls; however, it was approximately half of the mean level (18 900 pg/mL) in pregnant controls^32^ (table 3).

**Table 3:** ELISA of plasma ANGPT2 concentration

Individual	Gender	Mutation	ANGPT2 plasma concentration (pg/mL)	
Control 1	Female	–	1690	2196
Control 2	Female	–	1197	
Control 3	Male	–	2354	
Control 4	Female	–	1729	
Control 5	Female	–	1419	
Control 6	Male	–	2297	
Control 7	Male	–	1479	
Control 8	Female	–	2844	
Control 9	Female	–	4751	
LE-623-10*	Female	c.1475G>A	1247	
II-1	Male	c.557A>G	672	
II-2	Female	c.557A>G	8294†	

Created by the authors.

*Patient previously published.^23^

†The mother (II-2) was pregnant at the time of sampling.

ANGPT2, angiopoietin-2.

### Lymphohistochemical staining and placental examination

No obvious lymphatic abnormalities were noted, possibly due to substantial autolysis, which was present in tissue specimens from the affected fetuses. Placentas from pregnancies III-4 and III-5 were thick and oedematous (table 1). Abnormal maturation of chorionic villi was present in III-5.

## Discussion

Two of the authors recently reported heterozygous pathogenic sequence variants in *ANGPT2* as a cause of dominantly inherited primary lymphoedema.^24^ Here, we report a homozygous variant, NM_001147.2:c.557A>G r.557_566del, in a consanguineous family with recurrent, severe, early-onset hydrops fetalis. The unaffected parents and their two unaffected children are heterozygous and the four affected fetuses homozygous for this LOF variant.

### ANGPT2 c.557A>G activates a cryptic splice site and prompts nonsense-mediated mRNA decay, NMD/LOF

Rather than having an effect via an amino acid substitution, the variant activates a cryptic RNA splice site, predicted to remove 10 bp from the coding sequence, leading to a premature stop codon after the third of nine exons, (p.Asp186Valfs*3). Nonsense-mediated mRNA decay results in destruction of this mutant transcript. As confirmed in the unaffected parents, the loss of one allele is reflected by a halving of the expected amount of ANGPT2 plasma protein. ANGPT2 levels in the unaffected heterozygous children have not been assayed as their samples were unavailable. In the homozygous affected fetuses, there was likely a complete absence of ANGPT2 with resultant impaired lymphatic development and severe, lethal hydrops fetalis. Lymphatic dysplasia was not histologically evident in the skin and lung specimens from two affected fetuses. However, at the time of microscopy, autolysis was in process (data not shown). Taken together with earlier reports in humans and the murine model, we nonetheless believe that lymphatic dysplasia is the likely underlying cause of *ANGPT2*-related fetal hydrops.

### Angpt2 knockout mice have disturbed angiogenetic remodelling

The murine knockout mimics the severe phenotype described in this family. In the vascular system, ANGPT2 is considered an antagonist of ANGPT1-induced TIE1-TIE2 (tyrosine kinase with immunoglobulin-like and epidermal growth factor-like domains 1 and 2) signalling, while it seems to play a comparatively prominent activating role in lymphatic vasculature. *Angpt2* knockout mice have normal embryonal vascular development, but disrupted postnatal angiogenic remodelling.^26^ In addition, they exhibit major lymphatic vessel defects and generalised lymphatic dysfunction, including chylous ascites. Large lymphatic vessels are replaced by a ragged mesh, and small vessels exhibit abnormal patterning.^26^ The lymphatic phenotype, but not the blood vascular remodelling defects appearing later in development, can be rescued by expressing *Angpt1* instead of *Angpt2* in the *Angpt2* locus, showing an activating role of Angpt2 in lymphatics, and in contrast an antagonistic role in blood vessels.^26^ *ANGPT2* blocking antibodies also affect the formation of button-type lymphatic endothelial cell (LEC) junctions, adherence junction stability or lymphatic valve formation and maturation of collecting lymphatic vessels in mouse embryos.^33^ In contrast to the fetuses in our family who died before 27 weeks of gestation, mice homozygous for deletion of *Angpt2* were born at normal frequencies, but die from severe chylous ascites and other lymphatic defects by 2 weeks postnatally.^26^ Rare mice that survived to adulthood had severe ascites and lymph stasis.^26^

### Heterozygous ANGPT2 LOF mutations cause mild congenital lymphoedema

In the original report of seven affected individuals from five families with heterozygous *ANGPT2* mutations, the disorder was characterised by mild lymphoedema in early life, with resolution in three individuals during the first months after birth or adolescence and reduced penetrance in two unaffected mutation carriers.^24^ A heterozygous whole-gene deletion was identified in a 1.5-year-old boy followed for lower leg oedemas first noted at age 8–9 weeks.^24^ Three reported amino acid substitutions (Asn304Lys, Cys435Ser and Arg492Gln) resulted in LOF together with partially inhibited secretion of the WT allele in vitro, which may have a partial dominant-negative effect on ANGPT2 signalling.^24^ Variable effects on ANGPT2 are further exemplified by a fifth mutation (Thr299Met) that leads to gain-of-function, inducing increased lymphatic proliferation when expressed in the mouse ear.^24^ The associated clinical phenotype was progressive, hypothesised to be due to hyperplasia. However, genotype–phenotype correlations are tentative due to the low number of cases.^24^ These results indicate an optimal window of activity for ANGPT2, from which deviation upwards or downwards may significantly hamper development of lymphatic vasculature.

### Is there a lymphoedema phenotype in c.557A>G heterozygotes?

Heterozygosity for c.557A>G causes haploinsufficiency, and we aimed to delineate the phenotype of the heterozygotes in our family. In individual III-9, significantly increased nuchal translucency was detected on ultrasound at week 12 but was completely resolved 2 weeks later. Pregnancy was monitored closely, and there were no other signs of oedema in the fetus or the mother. The healthy newborn boy was not oedematous, and no oedemas developed by age 12 months. Heterozygosity for the pathogenic sequence variant might explain his transiently and significantly increased nuchal translucency; no other likely cause was found. Indeed, one of the individuals reported by Leppänen *et al* 24 also had increased nuchal translucency early in pregnancy. In our family, there is no evidence of primary lymphoedema in the heterozygous child nor in the parents, although pregnancy and neonatal records were not available for all. Thus, postnatal transient lymphoedema cannot be excluded. Furthermore, genetic predisposition may play a role when secondary lymphoedema develops in other contexts, for example, after surgery for breast cancer.^34 35^ Cellulitis is a well-recognised complication of primary lymphoedema.^36^ Whether or not clinically unaffected heterozygotes for a pathogenic *ANGPT2* variant are at increased risk of secondary lymphoedema and subsequently for cellulitis is not known.

### What is the cause of lymphoedema in individual II-6?

Lymphoscintigraphy of individual II-6 revealed lower extremity lymphoedema, abnormal morphology of lymphatic vessels in the right leg and absence of detectable lymphatic vessels in the left leg. Sanger sequencing of two separate blood samples and targeted NGS failed to identify the likely pathogenic variant in *ANGPT2* or any likely pathogenic variant in primary lymphoedema genes. Thus, the cause of her lymphoedema remains unknown.

### Phenotype in the affected fetuses

This family demonstrates uniquely the natural course of complete LOF of *ANGPT2* in humans. With one exception, the affected pregnancies were not terminated, but monitored until fetal demise. All three affected fetuses, for which a nuchal translucency measurement in the first trimester was available (III-5, III-7 and III-8), showed increased values, approaching what is seen in nuchal lymphatic malformations (cystic hygromas). Progression of hydrops was gradual from increased nuchal translucency to cystic hygroma, extensive subcutaneous fluid accumulation, hydrothorax, and lastly ascites, with intrauterine death occurring between weeks 14 and 27. The significantly, but transiently, increased nuchal translucency in a heterozygous unaffected fetus (III-9) underscores the importance of repeated ultrasounds in the context of increased nuchal translucency of unknown cause. We noted no specific feature that distinguishes *ANGPT2*-related hydrops from other forms of NIHF.

### Additional phenotypic features in the homozygous fetuses

Fetus III-4 had tetralogy of Fallot and fetus III-5 had a structurally normal heart. Two fetuses (III-7 and III-8) died too early to allow thorough assessment of cardiac anatomy. In a large cohort of individuals with tetralogy of Fallot, there was no increased burden of rare variants in *ANGPT2* (K Devriendt, MD, personal communication, December 2020). Thus, it is unclear whether congenital heart malformations are a feature of the homozygous *ANGPT2* phenotype. WES of fetus III-4 did not reveal any other pathogenic or likely pathogenic variants in genes known to be associated with tetralogy of Fallot.

Fetus III-5 revealed short long bones radiographically. This could have been due to general growth retardation via placental dysfunction or more specifically related to vascular/lymphatic abnormalities.

Fetus III-4 was probably growth retarded given the 2-week discrepancy between estimated due date based on last menstrual period versus sonographic estimation of gestational age. The length of the long bones was not commented on autopsy.

### ANGPT2 biallelic variants: a rare cause of hydrops fetalis

Biallelic LOF variants are likely a rare cause of recessive lymphatic-related hydrops fetalis. We are not aware of any other biallelic *ANGPT2* LOF variants in other cohorts of fetal hydrops to date, and no other match for *ANGPT2* was identified via GeneMatcher. The condition seems to be very severe, perhaps even universally lethal. Other cases may remain undiagnosed due to early fetal demise in the absence of molecular genetic investigations, as suggested by Quinn *et al*.^11^ In the same signalling pathway as *ANGPT2*, other genes have known phenotypes related to inherited monoallelic or somatic variants. Somatic and constitutional variants in the TIE2-encoding gene *TEK* cause venous malformations via a gain-of-function mechanism,^37–39^ while constitutional LOF variants may underlie primary congenital glaucoma.^40^ *ANGPT1* variants are reported in primary congenital glaucoma^41^ and hereditary angio-oedema.^42^ Homozygous LOF of *ANGPT1* and *TEK* is expected to be embryonically lethal in humans, as in mice, due to defective blood vessel formation,^43 44^ but not expected to cause fetal hydrops.

### WES in the diagnostic work-up of NIHF

The Society for Maternal-Fetal Medicine’s guidelines for the diagnostic work-up of NIHF from 2015^3^ does not address testing for monogenic disorders. However, given the rapid identification of new single gene disorders, WES is recommended as the preferred sequencing approach for NIHF.^11^ The likelihood of identifying new disease genes via single cases is high given the severity and decreased viability of the phenotype,^11^ as in this family.

## Conclusion

In conclusion, we report the first family with severe, early-onset hydrops fetalis caused by a homozygous LOF variant in *ANGPT2*. Our findings are in accordance with the previous identification of heterozygous missense variants causing autosomal dominant primary lymphoedema and with the crucial role of *ANGPT2* in lymphatic development demonstrated by the murine knockout model. Biallelic *ANGPT2* LOF is yet another of the many monogenic causes of NIHF. Subsequent studies may be able to confirm our suspicion of an underlying generalised lymphatic dysplasia. Heterozygous carriers of LOF variants may manifest signs of lymphatic insufficiency, such as dependent lymphoedema or transiently increased fetal nuchal translucency in early pregnancy.

## Data Availability

All data relevant to the study are included in the article or uploaded as supplementary information.
